# A *DNMT3B* Alternatively Spliced Exon and Encoded Peptide Are Novel Biomarkers of Human Pluripotent Stem Cells

**DOI:** 10.1371/journal.pone.0020663

**Published:** 2011-06-15

**Authors:** Sailesh Gopalakrishna-Pillai, Linda E. Iverson

**Affiliations:** Stem Cell Biology, Beckman Research Institute of City of Hope, Duarte, California, United States of America; University of Southern California, United States of America

## Abstract

A major obstacle in human stem cell research is the limited number of reagents capable of distinguishing pluripotent stem cells from partially differentiated or incompletely reprogrammed derivatives. Although human embryonic stem cells (hESCs) and induced pluripotent stem cells (iPSCs) express numerous alternatively spliced transcripts, little attention has been directed at developing splice variant-encoded protein isoforms as reagents for stem cell research. In this study, several genes encoding proteins involved in important signaling pathways were screened to detect alternatively spliced transcripts that exhibited differential expression in pluripotent stem cells (PSCs) relative to spontaneously differentiated cells (SDCs). Transcripts containing the alternatively spliced exon 10 of the *de novo* DNA methyltransferase gene, *DNMT3B*, were identified that are expressed in PSCs. To demonstrate the utility and superiority of splice variant specific reagents for stem cell research, a peptide encoded by *DNMT3B* exon 10 was used to generate an antibody, SG1. The SG1 antibody detects a single DNMT3B protein isoform that is expressed only in PSCs but not in SDCs. The SG1 antibody is also demonstrably superior to other antibodies at distinguishing PSCs from SDCs in mixed cultures containing both pluripotent stem cells and partially differentiated derivatives. The tightly controlled down regulation of *DNMT3B* exon 10 containing transcripts (and exon 10 encoded peptide) upon spontaneous differentiation of PSCs suggests that this DNMT3B splice isoform is characteristic of the pluripotent state. Alternatively spliced exons, and the proteins they encode, represent a vast untapped reservoir of novel biomarkers that can be used to develop superior reagents for stem cell research and to gain further insight into mechanisms controlling stem cell pluripotency.

## Introduction

Advancements in the studies of human embryonic stem cells (hESCs) and induced pluripotent stem cells (iPSCs) have created new opportunities for basic research and regenerative medicine [1]. These cells have wide-ranging applications in cell replacement therapies, development of model systems for studying diseases and drug testing. To realize the full potential of pluripotent stem cells (PSCs), however, many hurdles must be overcome. For example, PSCs propagated *in vitro* often spontaneously differentiate into unknown or undesired cells types. Although spontaneous differentiation of mouse ES cells can be prevented by supplementing the media with leukemia inhibitory factor (LIF), LIF does not prevent differentiation of human ES cells and comparable factors have not been identified [2]. In addition, limitations in the ability to detect dynamic changes in PSCs during self-renewal and early stages of differentiation are due primarily to a dearth of reliably accurate and sensitive detection assays. Additional reagents are needed to detect loss of pluripotency and to refine culture conditions that promote maintenance of the pluripotent state.

The transcriptional profiles of hESC and iPSC genes that regulate self-renewal, asymmetric cell division and signaling pathways are currently being characterized; however, relatively little is known about post-transcriptional gene regulatory mechanisms that operate in PSCs. Bioinformatic analysis of expressed sequence tags deposited in public databases indicate that hESCs express alternatively spliced variants of many genes that play important roles in signaling pathways that have been implicated in development and differentiation [3]. Hybridization of RNA isolated from hESCs and neural progenitors to exon microarrays identified several genes for which expression ratios of alternative splice variants differed during neural differentiation [4]. The widespread alternative splicing observed across various classes of hESC genes, including multiple components of signaling pathways, strongly suggests that alternative splicing is a key regulator of hESC gene expression. Despite these findings, little effort has been directed at investigating alternatively spliced variants as unique markers of pluripotency, specific differentiation stages or cell type lineages.

In this study, we demonstrate that several signaling pathway genes exhibit changes in alternative splicing patterns during the transition from PSCs to spontaneously differentiated cells (SDCs). Specific exons that were expressed at high levels in PSCs, but not expressed (or expressed at lower levels) in SDCs were identified. As proof-of-principle, one pluripotent stem cell-specific, alternatively spliced exon was used to generate a peptide-specific polyclonal antibody and shown to be an outstanding reagent for distinguishing human PSCs from cells undergoing early stages of spontaneous differentiation.

## Materials and Methods

### Pluripotent stem and differentiated cell culture

Karyotypically normal PSCs, including three hESC lines (H9 [WiCell], HES4 [IS], BG01 [Bresagen]) and the iPSC foreskin clone 1 (a generous gift from Dr. James Thomson, [5]), were maintained either on gamma-irradiated mouse embryonic fibroblasts feeder layer (CF-1, ATCC) or under feeder-independent conditions on matrigel coated dishes (BD) as described in detail previously [6] and briefly below. The hESCs were expanded on matrigel prior to harvesting RNA and protein to prevent any contamination from MEF-derived mouse gene products in molecular experiments. Media contained DMEM/F-12 with glutamine, 20% knockout serum replacement, 2 mM non-essential amino acids (all from Invitrogen) and 20 ng/ml zbFGF [7]. Cells were cultured under 5% CO_2_ at 37°C. For passaging, 5–6 day old hESC colonies were cut into small pieces (100–200 cells) by mechanical dissection using a 27G hypodermic needle and transferred to new dishes at a split ratio of 1∶3. To obtain spontaneously differentiated cells, undifferentiated PSC colonies grown on matrigel were fed with hESC media without zbFGF for the number of days indicated in each figure legend. Specifically, mixed cultures of PSCs and SDCs were produced by culturing in the absence of zbFGF for 4–5 days, while relatively homogeneous cultures of SDCs were obtained by maintaining in culture minus zbFGF for 14–15 days. Cultures of homogeneous SDCs (14–15 days minus zbFGF) were examined for any clusters of undifferentiated cells, which were removed from the dish prior to harvesting RNA or protein for molecular experiments.

### Semi-quantitative and realtime RT-PCR analysis

Total RNA was isolated from pluripotent stem and differentiated cells using the TRIzol method according to the manufacturer's (Invitrogen) instructions. To examine splice variant expression, cDNAs were synthesized from total RNA (2 µg) using the SuperScript II reverse transcriptase kit (Invitrogen) and random primers. PCR reactions were carried out using cDNA (1 µl) and exon-specific primers (Table S1) designed from information contained in alternative splicing databases such as Fast-DB (http://193.48.40.18/fastdb/), Ensembl (www.ensembl.org/index.html), Hollywood (http://hollywood.mit.edu/hollywood/) and UCSC genome browser (http://genome.ucsc.edu/). PCR products were resolved by electrophoresis on 1.5% agarose gels and visualized by ethidium bromide staining. Data were recorded using QuantityOne software (Biorad). PCR products of interest were excised, purified and directly sequenced or subcloned into pSC-A (Stratagene) and sequenced (Figure S1). Realtime RT-PCR was performed in duplicate for each sample in an iCycler (BioRad). Reactions (25 µl) contained cDNA template (1 µl), exon-specific primers and SYBR green PCR mix (Applied Biosystems). Relative quantification was done by the ΔΔCT method [8].

### Peptide antibody development

Open Biosystem (Huntsville, AL) synthesized the peptide, KSKVRRAGSRKLESR, encoded by *DNMT3B* exon 10, and produced the polyclonal antibodies. Two rabbits were injected with the above peptide conjugated to keyhole limpet hemocyanin. This study was carried out in strict accordance with the recommendations in the Guide for the Care and Use of Laboratory Animals of the National Institutes of Health. The protocol was approved by the Animal Care and Use Committee (IACUC) of Thermo Scientific, Open Biosystems (NIH (OLAW) assurance number: A3669-01; expires 03/31/2012; USDA (research license) registration number: 23-R-0089; expires 06/06/2011; PHS assurance number: A3669-01; expires 03/31/2012). Peptide antibody specificity was determined by ELISA and the affinity purified α-DNMT3B exon 10-encoded peptide-specific rabbit polyclonal antibody was designated SG1.

### Immunofluorescence staining of PSCs and SDCs

Cells were grown on matrigel-coated LabTek four or eight chamber slides, rinsed briefly with 1× PBS and fixed with 4% paraformaldehyde for 30 min at room temperature (RT). Samples were blocked with a solution containing 5% donkey serum and 5% Triton ×100 in 1× PBS for one hour at RT, then incubated with primary antibodies at 4°C overnight. Primary antibodies used included rabbit polyclonal α-OCT4 (1∶100, Cell Signaling, catalog # 2750), goat polyclonal α-OCT4 (1∶100, Santa Cruz, catalog # SC-8628), mouse monoclonal α-OCT4 (POU5F1; 1∶100, Sigma catalog # P0082), mouse monoclonal α-TRA-1-60 (1∶500, Cell Signaling, catalog # 4746), rabbit polyclonal α-DNMT3B (H-230, 1∶100, Santa Cruz, catalog # 20704), rabbit polyclonal α-DNMT3B (CS, 1∶100, Cell Signaling, catalog # 2161) and rabbit polyclonal SG1 (1∶100). After overnight incubation with primary antibody, slides were washed four times with 1× PBS, incubated with secondary antibodies for one hour at RT and washed four times with 1× PBS. Secondary antibodies, purchased from R&D system, included α-mouse IgG-NL493 (catalog # NL009), α-rabbit IgG-NL493 (catalog # NL006), α-goat IgG-NL493 (catalog # NL003), α-rabbit IgG-NL557 (catalog # NL004) and α-mouse IgG-NL557 (catalog # NL007). Secondary antibodies, purchased from Invitrogen, included α-mouse IgG-Alexa fluor 488 (catalog # 11017), α-mouse IgM-Alexa fluor 488 (catalog # A21042) and α-rabbbit IgG Alexa fluor 545 (catalog # A11071). All secondary antibodies were used at 1∶500 dilutions. Cells were counter-stained using Hoechst/1× PBS and coverslips mounted using Vectashield mounting medium. Fluorescence images were captured on an Olympus Inverted IX81 fluorescence microscope. All images of cells are shown at 100× magnification except as indicated in the figure legends.

### Western blots

Pluripotent and spontaneously differentiated cells were grown on six well matrigel-coated plates under feeder-independent conditions. Cells were rinsed twice with ice cold PBS and 0.5 to 1 ml of RIPA lysis buffer (Sigma) was added. Plates were kept at 4°C for 5 min. Lysate was clarified by centrifugation (10,000 g, 20 min), and was used immediately or stored at −80°C. Proteins were quantified using the BCA method (Pierce). Protein (15 µg/lane) was separated by electrophoresis on a 5–15% SDS polyacrylamide gel and blotted to a nitrocellulose filter using a semi-dry blotter apparatus (Bio-Rad). Primary antibodies used for Western blots were SG1 (1∶100), rabbit polyclonal α-OCT4 (1∶100) and mouse monoclonal α-GAPDH (1∶200, Santa Cruz, catalog # SC-47724). Secondary antibodies were horseradish peroxidase-coupled α-rabbit IgG (1∶10000; Santa Cruz) or α-mouse IgG (1∶10000; Santa Cruz). Secondary antibodies were detected using the ECL plus Western blotting detection system (GE Healthcare). For the dot blot assay, DNMT3B exon 10 peptide antigen was adsorbed to PVDF membrane (BioRad #162-0174) at decreasing concentrations, incubated with SG1 antibody (1∶100) or pre-immune sera (1∶100) followed by incubation with secondary antibody (horseradish peroxidase coupled α-rabbit IgG, 1∶10000, Santa Cruz), and the blot developed using the ECL plus Western blotting detection system.

## Results

### Alternative splice variants of signaling pathway genes are differentially expressed as pluripotent stem cells spontaneously differentiate

**Figure 1:**
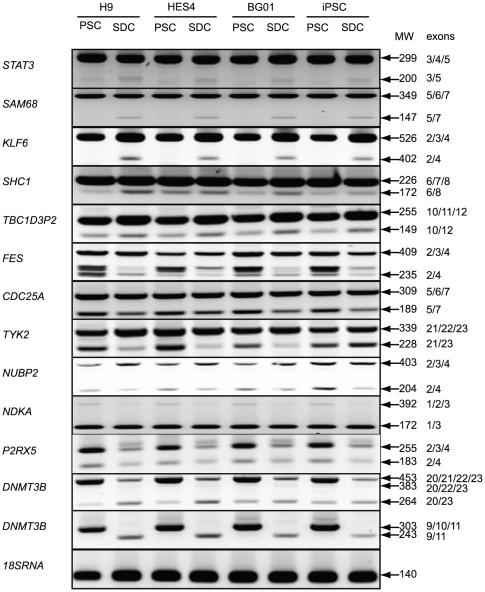
Human PSCs exhibit differential expression of alternative splice variants as the cells spontaneously differentiate. RT-PCR analysis using exon-specific primers was performed using total RNA isolated from pluripotent stem cells (PSCs) or cells that had undergone spontaneous differentiation for 14–15 days (SDCs). Gene names are on the left and PCR product size (MW) and exon identity (exon) are indicated on the right. 18sRNA was used as control in RT-PCR reactions.

To identify splice variants that displayed unique expression patterns in PSCs relative to SDCs, three hESC lines (H9, HES4, BG01) and one iPSC line (foreskin-1) were cultured under conditions that either maintained the pluripotent state or induced spontaneous differentiation. Genes chosen for examination included those with known or predicted splice variants that had been deposited in several alternative splicing databases (Materials and Methods) or alternatively spliced genes that had been implicated in stem cell differentiation in other organisms [3]. An emphasis was placed on genes encoding signaling proteins because of the probability that these genes are not simply markers of ‘stemness’ but play a functional role in maintaining the pluripotent state and, thus, may exhibit tighter regulation of splice variant expression as a function of pluripotency. Total RNA was extracted from PSCs and corresponding SDCs and subjected to semi-quantitative RT-PCR analysis using exon-specific primers. This analysis confirmed the existence of numerous alternatively spliced variants and revealed interesting changes in splicing patterns and expression ratios of splice isoforms as cells transitioned from the pluripotent to the spontaneously differentiated state (Figure 1).

The differential splicing patterns observed in PSCs relative to SDCs fell into four general categories. In the most prevalent category, the exon-excluded splice variant was expressed at higher levels in SDCs relative to PSCs. These genes included *STAT3* (*signal transducer and activator of transcription 3*), *SAM68* (*KH domain containing, RNA binding, signal transduction associated 1*), *KLF6* (*Kruppel-like factor 6*), *SHC1* (*SHC transforming protein 1*) and *TBC1D3P2* (*TBC1 domain family member 3, pseudogene 2*) (Figure 1). The second general category included those genes for which the exon-excluded variant was expressed at higher levels in PSCs than SDCs. Examples included *FES* (*feline sarcoma oncogene*), *CDC25A* (*cell division cycle 25 homolog A*) and *TYK2* (*tyrosine kinase 2*). In addition to precise exon skipping, some genes selected for comparison (e.g. *FES*), exhibited complex changes in alternative splicing pattern including generation of a novel splice isoform that arises by utilization of a 3′ acceptor site located within the downstream exon.

For many of the splice variants, the length of the excluded exon was a multiple of three, indicating that precise exon skipping preserved the open reading frame and suggesting that the excluded exons encode important protein structural, functional or regulatory domains. For the exon-excluded splice variants, one could design a peptide encoded by sequences spanning the exon junction and use this ‘exon junction’ peptide to raise antibodies that might distinguish between proteins translated from exon-excluded vs. exon-included transcripts; however, this approach is less straightforward than generating antibodies to peptides encoded by differentially included exons. Therefore, we were primarily interested in identifying splice variants in which the exon-included variant was expressed at higher levels in either PSCs or SDCs.

These splice variants were found in the two remaining general categories: those genes for which the exon-included variant was expressed at higher levels in SDCs than PSCs or those for which the exon-included variant was expressed at higher levels in PSCs relative to SDCs. Among the genes analyzed, the third category was least common. In general, exon-included variants were expressed at similar or higher levels in PSCs than SDCs, indicating that the frequency of exon skipping increases after differentiation or is concomitant with loss of pluripotency. Nonetheless, for *NUBP2* (*nucleotide binding protein 2*) the exon-included variant was expressed at higher levels in SDCs than PSCs (Figure 1). However, it is unlikely that a *NUBP2* exon 3 peptide-specific antibody would be a reliable marker of SDCs, since overall expression of *NUBP2* was low and its exon 3-included splice variant was also expressed in PSCs.

In the final category, the exon-included variant was expressed at higher levels in PSCs than SDCs. These genes included *NDKA* (*Nucleoside diphosphate kinase A*), *P2RX5* (*purinergic receptor P2X, ligand-gated ion channel 5*) and *DNMT3B* (*DNA cytosine-5-methyltransferase 3 beta*). In the case of *NDKA*, the exon 2-included variant was expressed at low levels in PSCs and disappears during spontaneous differentiation. Both the exon 3-included and exon 3-excluded variants of *P2RX5* were expressed at higher levels in PSCs compared to SDCs. In addition, an unknown *P2RX5* splice variant that migrated above the full-length product was specifically expressed in SDCs (Figure 1). Although these splice variants might be suitable for antibody production, the low level expression of the *NDKA* exon 2-included splice variant in PSCs and the fact that the *P2RX5* exon 3-included splice variant persisted in SDCs indicates that they are not ideal candidates for antibody production since protein expression levels may be too low to be detectable and/or expression is not unique to PSCs or SDCs.

**Figure 2:**
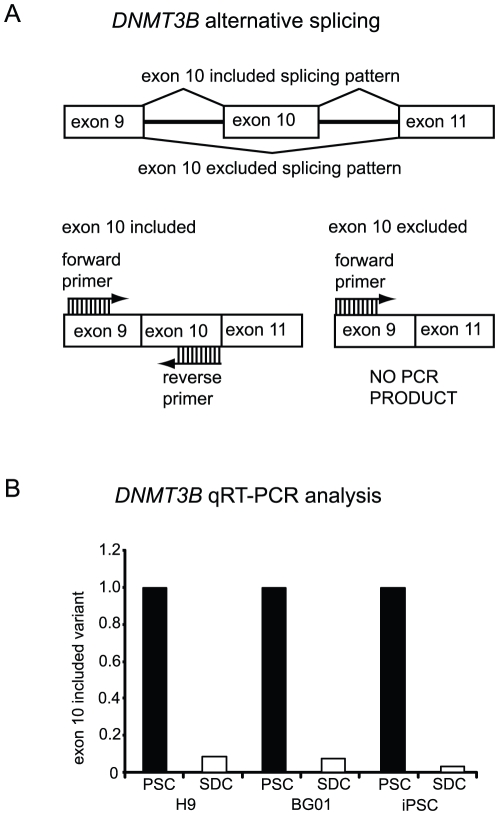
Exon 10-included DNMT3B splice variant is expressed at higher levels in pluripotent stem cells. A. Depiction of alternative splicing of *DNMT3B* exon 10 and location of PCR primers for qRT-PCR reactions. B. Quantitative changes in expression of *DNMT3B* exon 10, as measured by realtime PCR, in undifferentiated pluripotent stem cells (PSCs) or spontaneously differentiated cells (SDCs; 14–15 days), in the H9 hESC, BG01 hESC and foreskin-1 iPSC lines.

However, RT-PCR analysis of *DNMT3B* splice variants in PSCs relative to SDCs indicated that *DNMT3B* encodes at least one ideal candidate for antibody production. Two primer pairs were used to analyze expression patterns of *DNMT3B* splice variants. In one pair, an exon 20 forward primer was used in conjunction with an exon 23 reverse primer to examine differential expression of the DNMT3B catalytic domain encoded by exons 21 and 22. The full-length isoform was predominately expressed in PSCs, and its expression decreased, but did not disappear, during the transition from PSCs to SDCs. *DNMT3B* transcripts lacking exons 21 and 22 were also detected in PSCs and their expression level increased upon differentiation (Figure 1). In contrast, the primer pair specific for exons 9 (forward) and 11 (reverse) amplified a major *DNMT3B* transcript containing exon 10 that was specifically expressed in PSCs and absent from SDCs. A similar finding was reported for mouse ES cells where undifferentiated cells express *DNMT3B* transcripts that include exon 10, while exon 10 is excluded from *DNMT3B* transcripts in differentiated cells [9]. To confirm that *DNMT3B* exon 10 was uniquely expressed in PSCs, realtime RT-PCR analysis was performed with an exon 9 forward primer and an exon 10 reverse primer (Figure 2A) using RNA extracted from three pluripotent stem cell lines (H9, BG01 and iPSC) and their respective spontaneously differentiated derivates. *DNMT3B* exon 10 expression levels were 11-fold higher in H9 PSCs vs. H9 SDCs, 13-fold higher in BG01 PSCs vs. BG01 SDCs and 32-fold higher in iPSCs vs. iPSC SDCs (Figure 2B).

### Peptides encoded by alternatively spliced exons can be used to raise antibodies that distinguish pluripotent stem cells from early stage differentiated cells

**Figure 3:**
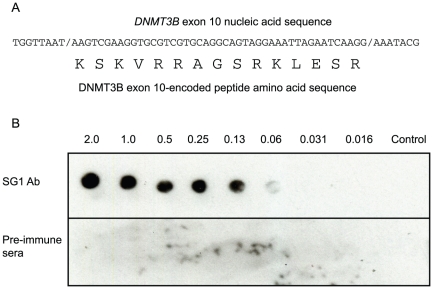
DNMT3B exon 10 and encoded peptide sequence used for immunization. A. Sequence of *DNMT3B* exon 10 encoding a 15 amino acid peptide used for generating the SG1 peptide antibody. B. Dot blot analysis demonstrating specificity of SG1 antibody relative to pre-immune sera. Decreasing quantities (in µg) of peptide antigen were adsorbed to the membrane, incubated with either the SG1 antibody or pre-immune sera, incubated with secondary antibody and the blot developed as described in Materials and Methods. Pre-immune sera detects no peptide antigen even at the highest concentration.

Given the abundant, restricted expression of *DNMT3B* exon 10-included transcripts in PSCs relative to SDCs, this sequence was selected for peptide-specific antibody production. The peptide sequence was designed on the basis of the human *DNMT3B* exon 10 genomic sequence (Figure 3A). Sequence alignment using BLAST confirmed this sequence was specific to *DNMT3B* exon 10 and BLASTP confirmed the peptide sequence was unique. The 15 amino acid peptide was synthesized and used as antigen for immunization of rabbits. Specificity of the SG1 antibody relative to pre-immune sera was confirmed by performing dot blot analysis against decreasing concentrations of the peptide antigen used for immunization (Figure 3B).

**Figure 4:**
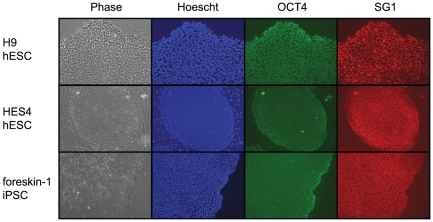
DNMT3B exon 10 encoded peptide antibody, SG1, detects pluripotent stem cells. Dual immunofluorescence assay of undifferentiated pluripotent stem cell lines, H9, HES4 and iPSC stained with OCT4 or SG1 antibodies. Phase contrast image of stem cell colonies (Phase) and same colony stained with Hoechst dye (Hoechst; blue), OCT4 antibody (OCT4; green), and SG1 antibody (SG1; red).

The affinity purified α-DNMT3B exon 10 encoded peptide polyclonal antibody, SG1, was first tested for its ability to detect DNMT3B expression in PSCs. Three undifferentiated pluripotent stem cell lines (H9, HES4 and iPSC) were cultured for 5–6 days and immunofluorescence staining was performed using a dual staining procedure to identify cells that stained positive with OCT4 and/or SG1 antibodies. OCT4 was chosen for comparison because it is considered a definitive marker of pluripotency. Complete overlap of OCT4 and SG1 staining was observed in both hESCs and iPSCs (Figure 4), indicting that the SG1 antibody identifies pluripotent stem cells.

**Figure 5:**
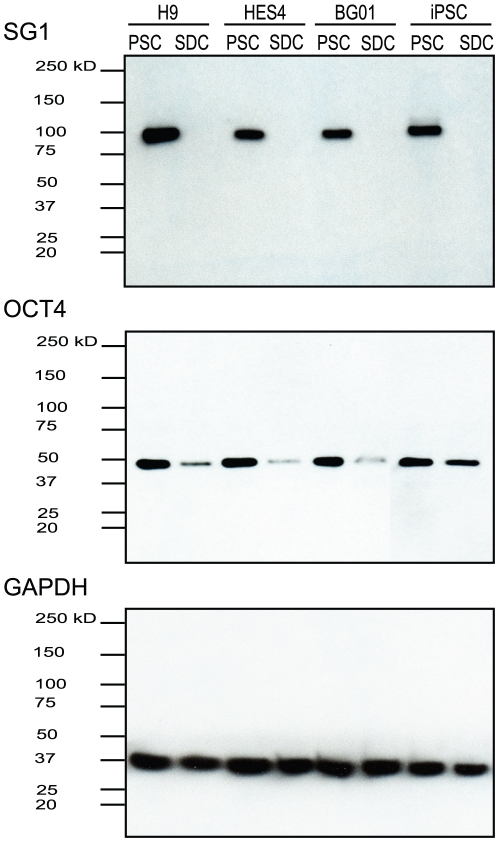
DNMT3B protein containing the exon 10-encoded peptide is expressed only in pluripotent stem cells. Western blot analysis using *DNMT3B* exon 10 peptide antibody (SG1), OCT4 and GAPDH (control) antibodies to detect proteins expressed in pluripotent stem (PSCs) and spontaneously differentiated cells (SDCs; 14–15 days).

We then asked if the SG1 antibody detects DNMT3B protein on Western blots of proteins extracted from four PSC lines (H9, HES4, BG01 and iPSC) and their corresponding spontaneously differentiated derivatives (14–15 days minus zbFGF). The SG1 antibody detected high-level expression of a single band of the expected MW (∼100 kD) that was present in all four PSC lines, but did not detect any protein in any of the four SDC populations (Figure 5). In contrast, low-level expression of OCT4 protein was detected in SDCs of all three hESC lines examined. Interestingly, OCT4 expression remained fairly high in SDCs derived from the iPSC line, suggesting that the *OCT4* transgene used to create this iPSC line may still be expressed even after differentiation.

**Figure 6:**
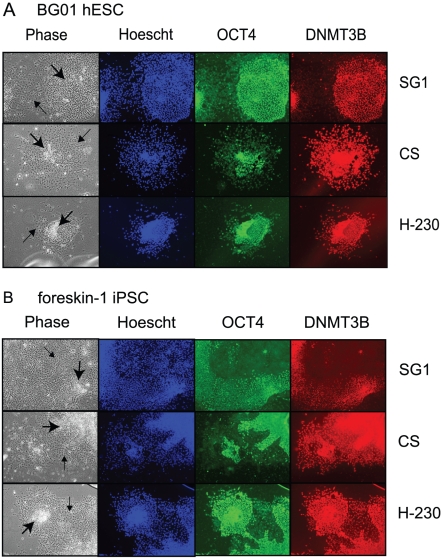
SG1 antibody identifies pluripotent stem cells in mixed populations. Mixed populations of pluripotent and early-stage spontaneously differentiated cells (4–5 days minus zbFGF) obtained from (A) BG01 hESC line or (B) foreskin-1 iPSC line were stained with SG1 antibody and compared to cells stained with α-OCT4 polyclonal and two commercially available α-DNMT3B polyclonal antibodies, one from Cell Signaling (CS) and one from Santa Cruz (H-230). Phase contrast image of stem cell colonies (Phase) and same cells stained with Hoechst dye (Hoecsht; blue), α-OCT4 antibody (OCT4; green) and one of three different α-DNMT3B antibodies (DNMT3B; red) as indicated on the right. The α-DNMT3B antibodies used included the custom peptide antibody, SG1 (top), or one of two commercial antibodies, CS (middle) or H-230 (bottom). Compact colonies of pluripotent stem cells are indicated by large arrows, while dispersed spontaneously differentiated cells are indicated by small arrows in the phase contrast images.

To confirm the utility of the SG1 antibody at distinguishing PSCs from SDCs in mixed populations containing both pluripotent stem cells and spontaneously differentiated derivatives, two pluripotent stem cell lines (BG01 and iPSC) were examined for SG1 staining while the cells were undergoing early stages of spontaneous differentiation. Undifferentiated PSC colonies were grown for four to five days in stem cell media in the absence of zbFGF until SDCs appeared. Immunofluorescence staining of these partially differentiated colonies was performed using the SG1 antibody in comparison to the α-OCT4 and two commercially available rabbit polyclonal α-DNMT3B antibodies. Low-level OCT4 expression was detected in SDCs derived from the BG01 hESC line. Neither DNMT3B commercial antibody distinguished PSCs from SDCs (Figure 6A). Similar results were obtained using mixed populations of PSCs and SDCs derived from the iPSC line (Figure 6B). In marked contrast, the custom α-DNMT3B peptide antibody, SG1, was highly specific to PSCs and did not stain SDCs in mixed populations of either the BG01 hESC (Figure 6A) or the iPSC (Figure 6B) lines.

**Figure 7:**
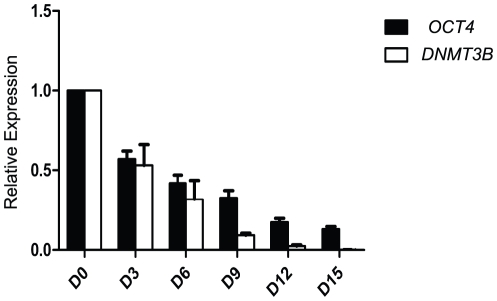
Time course of expression of DNMT3B exon 10 containing transcripts relative to OCT4 transcripts in spontaneously differentiating cells. RNA extracted from H9 cells induced to differentiate by removal of zbFGF from the media for the indicated number of days (0, 3, 6, 9, 12 or 15) was subjected to qRT-PCR analysis. Relative expression levels of *OCT4* transcripts (black bars) in comparison to *DNMT3B* exon 10 containing transcripts (white bars) are plotted as a function of the number of days of spontaneous differentiation. Duplicate qRT-PCR experiments were performed for each sample; the mean of the two experiments is plotted with SEM indicated by the error bars.

These results indicate that the SG1 antibody detects a unique DNMT3B protein isoform exhibiting an expression profile that is restricted to pluripotent stem cells. Given that expression of the DNMT3B protein isoform is down regulated faster than OCT4 protein upon spontaneous differentiation of pluripotent stem cells, we than asked if transcripts containing *DNMT3B* exon 10 also exhibit more tightly restricted expression than *OCT4* transcripts in cells undergoing early stages of spontaneous differentiation. For this experiment, we compared the time course of down regulation of *DNMT3B* exon 10 included transcripts relative to *OCT4* transcripts by quantitative RT-PCR analysis. H9 hESCs were induced to spontaneously differentiate by removal of zbFGF from the culture media. Cells were harvested at days 0, 3, 6, 9, 12 and 15, RNA extracted and qRT-PCR analysis performed using primer pairs specific for *DNMT3B* exon 10 (as depicted in Figure 2A and shown in Table S1) or *OCT4* (Table S1). Relative transcript expression levels were plotted as a function of number of days following induction of differentiation (Figure 7). The time course experiment demonstrates that *DNMT3B* exon 10 containing transcripts also exhibit faster down regulation than *OCT4* transcripts upon spontaneous differentiation. By day 6, which corresponds to early stages of spontaneous differentiation, *OCT4* transcripts in SDCs are expressed at levels equivalent to 42% of that detected in PSCs, while *DNMT3B* exon 10 transcripts have been reduced to 32% of the original level in PSCs; by day 15 (late stage spontaneous differentiation) *OCT4* transcripts in SDCs are still expressed at levels as high as about 13% the original level in PSCs, while expression levels of *DNMT3B* exon 10 transcripts have decreased significantly and are now expressed at levels less than 0.2% the original level observed in PSCs.

**Figure 8:**
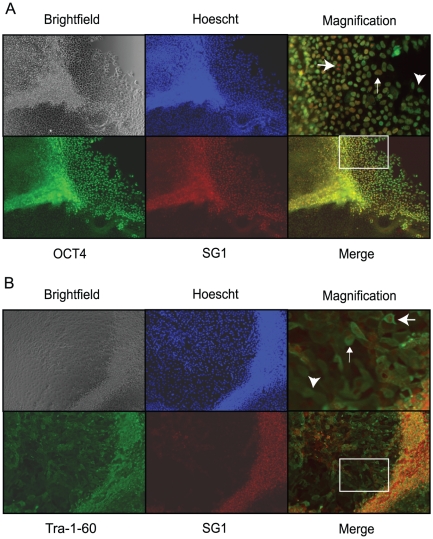
SG1 antibody is superior to OCT4 and TRA-1-60 antibodies at identifying pluripotent stem cells in mixed populations. Mixed populations of pluripotent and spontaneously differentiated cells (4–5 days minus zbFGF) obtained from the H9 hESC line were analyzed by dual immunofluorescence staining using SG1 rabbit polyclonal antibody and mouse monoclonal antibodies to OCT4 (A) or TRA-1-60 (B). A. Brightfield images of stem cell colonies (Brightfield) and same cells stained with Hoechst dye (Hoecsht; blue), α-OCT4 antibody (OCT4; green) and α-DNMT3B exon 10 encoded peptide (SG1; red) are shown. OCT4 and SG1 staining patterns are overlaid (Merge) and the area outlined by the white box in the Merge panel is shown in the Magnification panel to facilitate visualization of precise staining patterns in individual cells. The large arrow in the Magnification panel indicates a cell exhibiting high level expression of both OCT4 and SG1 (in this case, the cell is undergoing mitosis and the SG1 antibody ‘paints’ the chromatids of the dividing cell), the small arrow identifies a cell exhibiting high OCT4 but low SG1 staining, while the large arrowhead indicates a cell still expressing high levels of OCT4 that is not stained by the SG1 antibody. B. Similar analysis as in A (above) using a monoclonal antibody that detects the stem cell marker TRA-1-60 (green). As opposed to the intracellular proteins (above), the TRA-1-60 antibody detects the expected TRA-1-60 expression on the cell surface. While high-level TRA-1-60 expression is detected on almost all cells (both PSCs and SDCs), SG1 staining is more tightly restricted to PSCs or those cells in very early stages of spontaneous differentiation. All images are shown at 100× magnification with the exception of the two “Magnification” panels, which were increased in size by about 12 fold in order to allow visualization of individual cells.

To further confirm the observed faster down regulation of the DNMT3B exon 10 encoded peptide antigen relative to other protein biomarkers of pluripotent stem cells, we reexamined protein expression in mixed cultures of PSCs and SDCs using monoclonal antibodies detecting stem cell markers OCT4 and TRA-1-60 and compared their expression to that of the DNMT3B exon 10 encoded peptide antigen detected by the SG1 rabbit polyclonal antibody. H9 PSC colonies were grown in stem cell media in the absence of zbFGF for four to five days until SDCs appeared. Dual staining for the intracellular markers OCT4 and DNMT3B in mixed cultures undergoing early stage differentiation demonstrates that every cell that is detected by the SG1 antibody is also detected by the OCT4 antibody, indicating that DNMT3B exon 10 encoded peptide expression is restricted to those cells that express high-level OCT4 protein (Figure 8A). The converse, however, is not true. A number of cells, particularly those at a distance from the main colony, exhibit high-level OCT4 expression but are not stained by the SG1 antibody, indicating that DNMT3B exon 10 encoded peptide expression is tightly restricted to PSCs, while OCT4 protein expression persists in early-stage SDCs. Similar results were obtained when using a dual staining procedure to compare DNMT3B exon 10 encoded peptide expression relative to the cell surface expressed stem cell marker, TRA-1-60 (Figure 8B). Again, every cell detected by the SG1 antibody is also detected by the TRA-1-60 monoclonal antibody, while numerous cells, particularly those at a distance from the main colony, are stained by the TRA-1-60 antibody but not by the SG1 antibody, indicating that DNMT3B exon 10 encoded peptide expression is tightly restricted to PSCs, while TRA-1-60 protein expression persists in early-stage SDCs.

## Discussion

Among the many bottlenecks in human PSC research are an inability to prevent spontaneous differentiation of the cells in culture and the lack of robust, reliably specific reagents that distinguish PSCs from SDCs. Properties such as the ability to self renew or differentiate into cells of all three lineages are hallmarks of pluripotent stem cells that are controlled by exquisite gene regulatory mechanisms that operate at multiple levels, including transcriptional, post-transcriptional, translational and post-translational. Although more than 70% of human genes are alternatively spliced and alternative splicing is a major source of generating proteome diversity [10], its importance in stem cell research has been underappreciated and, to some extent, unrecognized. Rather than relying on differences in whole gene transcription to identify new markers of pluripotency, our approach relied on identifying alternatively spliced, protein-coding exons that were abundantly and uniquely expressed in PSCs. We focused on a few genes encoding components of signaling pathways that had been previously implicated in stem cell maintenance and differentiation because we suspected their alternative splicing patterns would be tightly regulated between the pluripotent and the spontaneously differentiated states.

Three hESC lines (H9, HES4 and BG01) and one human iPSC line (foreskin-1) were included in our study. Differential expression of alternatively spliced exons was functionally validated by RT-PCR and, in some cases, by direct sequencing. One particularly promising candidate, *DNMT3B* exon 10, was selected for generation of a peptide-specific polyclonal antibody, SG1. Restricted expression of *DNMT3B* exon 10 and the DNMT3B exon 10-encoded peptide to PSCs was confirmed by both qRT-PCR and Western blot analyses. The ability of the SG1 antibody to distinguish PSCs from SDCs was also compared to several commercially available polyclonal and monoclonal antibodies detecting stem cell proteins OCT4 and TRA-1-60. In every case, the *DNMT3B* exon 10-encoded peptide exhibited expression that was more restricted to PSCs. Because *OCT4* transcripts and OCT4 protein expression are currently considered the ‘gold standard’ for identification of pluripotent stem cells [11], our results indicate that *DNMT3B* alternatively spliced exon 10 and the SG1 antibody are superior reagents for distinguishing PSCs from partially differentiated derivates and can be used to better monitor the progressive loss of ‘stemness’ as hESCs differentiate, or the progressive gain of ‘pluripotency’ during nuclear reprogramming of iPSCs.

*DNMT3B* is a member of the DNA methyltransferase family that was identified as a *de novo* methylation agent of the human genome. The human *DNMT3B* gene encodes as many as 40 different isoforms through alternative splicing of *DNMT3B* transcripts. Various *DNMT3B* splice isoforms are highly expressed in the human female germ line, preimplantation embryos, and embryonic stem cells, and are differentially expressed during development and tumorigenesis [12], [13], [14]. *DNMT3B* was identified as a commonly overexpressed marker of 59 hESC lines by microarray analysis [15]; however, uniquely expressed splice variants are not generally detectable using conventional cDNA microarrays. *DNMT3B* was also suggested to be a specific marker of *bona fide* human pluripotent stem cells based on qRT-PCR analysis that did demonstrate a high degree of specificity of expression of *DNMT3B* transcripts in PSCs relative to partially reprogrammed cells [16]. This result, however, can be explained by the fortuitous choice of a PCR forward primer containing only *DNMT3B* exon 10 sequences. Therefore, it is important to recognize that not all *DNMT3B* transcripts or DNMT3B protein isoforms are unique and reliable markers of pluripotent stem cells.

DNMT3A and DNMT3B are two major *de novo* DNA methyltransferases. Loss of one or both results in abnormal global DNA methylation patterns; however, loss of DNMT3B (unlike DNMT3A) also results in hypomethylation of centromeric and pericentromeric satellite regions that leads to centromeric instability and mitotic defects [17]. Although the precise function of the *DNMT3B* exon 10-encoded peptide domain remains unknown, it lies between the PWWP and the ring-type zinc finger domains suggesting that it may play a role in modulating protein-protein interactions important for DNMT3B binding to H4K20me and/or targeting of DNMT3B to particular chromosomal sites [9], [18]. A series of recent reports indicate that gene expression profiles of iPSCs and hESCs are non-identical and that some iPSCs retain an epigenetic memory of their cell type of origin that could arise from distinct global and/or gene–specific DNA methylation patterns [19], [20], [21], [22], [23], [24], [25]. Furthermore, recent evidence indicates that the Werner Syndrome gene product, WRNp, localizes to the *OCT4* promoter of human PSCs undergoing retinoic acid induced differentiation where it plays a role in *de novo* DNA methylation by recruiting DNMT3B to the *OCT4* promoter [26]. These results raise the intriguing hypothesis that proteins encoded by *DNMT3B* exon 10-containing transcripts may play a crucial role in establishing *de novo* DNA methylation patterns that are characteristic of the pluripotent state perhaps by regulating transcription of the pluripotency transcription factor, OCT4, and, in so doing, might affect the efficiency and/or stability of nuclear reprogrammed iPSCs. Finally, the previously noted similarities in pluripotent and cancer stem cell gene expression patterns [27] suggest that *DNMT3B* exon 10 may be a specific biomarker of the stem cell component of some tumors.

## Supporting Information

Figure S1 Sequences of PCR amplified products, which were either sequenced directly using exon-specific primers or subcloned into StrataClone vector and sequenced using T3 primers.
(EPS)

Table S1 Sequences of exon-specific primers used for semi-quantitative RT-PCR (Figure 1) or real time PCR analysis (Figures 2 and 7).
(DOC)
