# Supplementary material for: A DNMT3B Alternatively Spliced Exon and Encoded Peptide Are Novel Biomarkers of Human Pluripotent Stem Cells
Source: PLoS One. 2011 Jun 15;6(6):e20663. doi: 10.1371/journal.pone.0020663 (PMC3115941; doi:10.1371/journal.pone.0020663)
Supplement: Table S1 — Sequences of exon-specific primers used for semi-quantitative RT-PCR (Figure 1) or real time PCR analysis (Figures 2 and 7). (DOC) [file pone.0020663.s002.doc]

| Gene | Forward Primer | Reverse Primer |
| --- | --- | --- |
|  | Semi-quantitative RT-PCR |  |
| *NUBP2*  (2/3/4) | CCTGGCCGGCGTCAGGCACATC | GGCCATGTGCTCATCGGAGGTC |
| *P2RX5*  (2/3/4) | CCCTGCAGAGTGCTGTCATCAC | CGTTTCCAGCTGTAACCGCTTC |
| *TBC1D3P2*  (10/11/12) | TTCACAGCCCAAATGGCGGGACC | GCATGAACGCCTGTTCGCCTTC |
| *FES*  (2/3/4) | GCTTCGTCTACTGGAGGGCATGAG | GGCCTCCTGGTACTTGCGCTTGGC |
| *SHC1*  (6/7/8) | CCCTGTTAGCCGCCCGCTCAGCTCT | CTCTCTGATTCACAGGGTCTTTGG |
| *CDC25A*  (5/6/7) | CAGAAGCTGTTGGGATGTAGTCC | AGACAAAGTGGCTGTCACAGGTG |
| *TYK2*  (21/22/23) | GAGTGCCTGAAGGAGTATAAGTT | CACTGAAGGGGCCTGGCCTTGGT |
| *STAT3*  (3/4/5) | CCACTTTGGTGTTTCATAATCTCC | CCGGACATCCTGAAGGTGCTGCTC |
| *SAM68*  (5/6/7) | GAGTACCTGAACCCTCTCGTGGACG | CCTTGACTCTGGCTGTAATAGCCTTCG |
| *NDKA*  (1/2/3) | CGTGCAAGTGCTGCGAACCACG | CGCTTGATAATCTCTCCCACAAG |
| *KLF6*  (2/3/4) | GCACCGGTGCCACTTTAACGGCTGCAGG | CTCCTTCCACGGCCGGCTCTCAGCCTGG |
| *DNMT3B*  (9/10/11) | GCTAGGGTGCGAGCTGGCAAGACC | GTCTTTGCCGTTGTTATAGCAATTTGTC |
| *DNMT3B*  (19/20/21/22/23) | GGCCGTTCTTCTGGATGTTTGAGAATG | GTCGGATGACAGGCACGCTCCAGGACC |
| *18S RNA* | CGGGCGCCGGCGGCTTTGGTGAC | GGTCACCATGGTAGGCACGGCGAC |
|  | Real time RT-PCR |  |
| *DNMT3B*  (9/10) | GCTAGGGTGCGAGCTGGCAAGACC | TAATTTCCTACTGCCTGCACGACGCACC |
| *OCT4* | CAGTGCCCGAAACCCACAC | GGAGACCCAGCAGCCTCAAA |
| 18S RNA | GGCACTTTCGATGGTAGTCGC | CGTTTCTCAGGCTCCCTCTCC |
